# Complete chloroplast genome of *Euphorbia resinifera*: overcoming biogeographical bias in phylogenetic inference and establishing a conservation genomics framework for threatened North-West African cactiform species

**DOI:** 10.3389/fpls.2026.1785579

**Published:** 2026-04-01

**Authors:** Abdelhakim Taha, Karim Rabeh, Mebarek Lamara, Fatima Gaboun, Ilham Laadsi, Nezha Lebkiri, Ghizlane Diria, Rabha Abdelwahd, Karim Saghir, Abderrahim Ettaqy, Mohamed El-Mderssa, Hamid Khamar, Matthew G. Johnson, Mohamed Fokar, Younes Abbas

**Affiliations:** 1 Sultan Moulay Slimane University, Polydisciplinary Faculty of Beni Mellal, Applied Biology for Health, Environment and Sustainable Development Laboratory, Beni Mellal, Morocco; 2 Oasis System Research Unit, Regional Center of Agricultural Research of Errachidia, National Institute of Agricultural Research, Errachidia, Morocco; 3 Institut de recherche sur les forêts, Université du Québec en Abitibi-Témiscamingue, Rouyn-Noranda, QC, Canada; 4 Biotechnology Unit, Regional Center of Agricultural Research of Rabat, National Institute of Agricultural Research, Rabat, Morocco; 5 Center for Biotechnology and Genomics, Texas Tech University, Lubbock, TX, United States; 6 Department of Botany and Plant Ecology, Scientific Institute, Mohammed V University, Rabat, Morocco; 7 Department of Biological Sciences, Texas Tech University, Lubbock, TX, United States

**Keywords:** *Euphorbia resinifera*, complete chloroplast genome, genome skimming, cactiform clade, *rpl22* gene, phylogenetic analysis, cpSSR analysis, plant systematics

## Abstract

**Introduction:**

*Euphorbia resinifera* O. Berg (1863) is an endemic cactiform species from Morocco's arid High Atlas Mountains, valued for its medicinal properties and melliferous importance. It faces conservation challenges from habitat degradation and phylogenetic incongruence between xeromorphic adaptations and current sectional classification.

**Methods:**

We pioneered a non-destructive spine-DNA genome-skimming approach to generate the complete chloroplast genome, and applied a multi-pronged molecular strategy including cpSSR profiling, whole-plastome phylogenetics, and *rpl22* gene analysis.

**Results:**

The complete chloroplast genome (163,065 bp; 35.11% GC) contains 132 genes and 96 SSR loci, representing the first genomic resource for threatened North-West African cactiform *Euphorbia*. cpSSR profiles recovered relationships only within sect. *Euphorbia*, while whole-plastome analysis showed biogeographical clustering. The *rpl22* gene precisely distinguished growth forms and revealed perfect sequence identity between morphologically similar pairs (*E. ampliphylla* and *E. resinifera, E. neospinescens* and *E. neoarborescens, E. micractina* and *E. pekinensis*), with *E. royleana* showing near-perfect identity to *E. ampliphylla* and *E. resinifera* (1 SNP difference). Notably, *E. drupifera* was excluded from the cactiform core within sect. *Euphorbia* through 10 diagnostic SNPs and a unique AGC deletion.

**Discussion:**

This study establishes *rpl22* as a powerful taxonomic discriminator for *Euphorbia* systematics, with future multi-gene approaches recommended for broader phylogenetic resolution.

## 1 Introduction

The genus *Euphorbia*, with over 2,000 species, constitutes the second largest angiosperm genus and forms a monophyletic group subdivided into four distinct subgenera (Wei et al., 2021). The subgenus *Euphorbia* comprises 21 sections, with sect. *Euphorbia* being the most morphologically variable, including over 340 species across Africa and South to Southeast Asia. Recent molecular phylogenetic studies using chloroplast markers (*matK, ndhF, rbcL, rpl16, trnL-F*) combined with nuclear ITS sequences have significantly advanced sectional classification within subgenus *Euphorbia* (Bruyns et al., 2006; Horn et al., 2012; Dorsey et al., 2013). However, species delimitation within sect. *Euphorbia* remains challenging due to conflicting morphological and molecular evidence, particularly where current phylogenetic markers fail to separate cactiform from pachycaul growth forms, as demonstrated by the problematic placement of pachycaul *E. drupifera* among morphologically distinct cactiform lineages (Horn et al., 2012; Dorsey et al., 2013).

Within sect. *Euphorbia*, cactiform xerophytes with ribbed stems represent a morphologically distinct group that resembles columnar Cactaceae but is distinguished by its latex secretion, cyathia inflorescences, and paired spines (Horn et al., 2012; Dorsey et al., 2013; Chaudhary et al., 2023). These species originated in Africa and occupy diverse habitats ranging from arid and semi-arid to humid tropical environments, most often on rocky or sandy soils (Horn et al., 2012, 2014; Ettaqy et al., 2020). North-west African species (*E. resinifera, E. officinarum, E. canariensis, E. handiensis*) share ecological and phylogenetic traits with East African and Arabian Peninsula taxa (Dorsey et al., 2013). Notably, *E. makallensis*, the East African cactiform most morphologically similar to North-West African *E. resinifera*, is of very limited distribution and declining in its native Ethiopian highlands (Wilson and Munro, 2019). Most xerophytic cactiform *Euphorbia* are declining in arid to hyperarid zones characterized by prolonged drought periods (Masrahi et al., 2015; Al-Qthanin and Alharbi, 2023; Taha et al., 2023).

*Euphorbia resinifera* O. Berg (1863), endemic to Morocco’s High Atlas and Anti-Atlas Mountains, exemplifies these taxonomic uncertainties. This multipurpose species, valued for medicinal properties and apiculture, has undergone rapid decline over recent decades, driven by increasing aridity and direct human pressure, potentially leading to extinction (Taha et al., 2023, 2024). It is currently classified as “Near Threatened” in Morocco’s Red Book of Vascular Flora (Fennane, 2018). Traditional tissue sampling approaches are problematic for cactiform species due to their leafless morphology and irritant latex, with permission to sample tissue particularly difficult to obtain for rare and threatened species held in living collections. However, spine-derived DNA extraction offers a practical non-destructive alternative for genomic studies of threatened succulent species (Fehlberg et al., 2013). Genome-skimming approaches are particularly effective for organellar genome recovery (Straub et al., 2012) and well-suited for degraded DNA samples (Li et al., 2021).

The chloroplast genome is a powerful tool for plant phylogenomics due to its conserved quadripartite structure, moderate substitution rates, maternal inheritance, and rare recombination events (Ruhlman and Jansen, 2014; Zhao et al., 2024). Within slow-evolving plastomes, chloroplast simple sequence repeats (cpSSRs) provide particularly valuable taxonomic signatures. Furthermore, cpSSR distribution patterns including their abundance, density, and GC content exhibit lineage-specific signatures that reflect underlying taxonomic relationships (Zhu et al., 2021). Chloroplast SSRs have proven particularly effective for resolving species relationships and characterizing phylogenetic patterns within complex plant genera (Vieira et al., 2016). Recent advances in plastome-based systematics have demonstrated the effectiveness of integrating complete genome sequences with targeted marker analysis for resolving complex evolutionary relationships across diverse angiosperm families (Kan et al., 2024; Dong et al., 2025).

To address conservation needs and clarify the taxonomic position of these threatened species, we sequenced and characterized the complete chloroplast genome of *E. resinifera* using a genome-skimming approach applied to DNA extracted from plant spines, the first documented non-destructive plastome sequencing protocol for cactiform *Euphorbia* species. Specifically, our objectives were to: (1) generate and annotate the first complete chloroplast genome of a leafless shrub cactiform *Euphorbia* with ribbed stem from the arid zones of North-West Africa and Arabia; (2) examine plastome structural features and IR/SC boundary dynamics within section *Euphorbia*; (3) characterize SSR abundance and distribution patterns across the chloroplast genome; and (4) identify targeted plastid loci with high taxonomic resolution for distinguishing cactiform xerophyte lineages from pachycaul species. Together, these results provide a conservation-friendly systematic framework for resolving taxonomic relationships and supporting molecular monitoring of threatened cactiform *Euphorbia* lineages from arid and hyperarid regions.

## 2 Materials and methods

### 2.1 Plant material collection and genomic DNA extraction

Due to its high latex content, *Euphorbia resinifera* shows poor regenerative capacity and is undergoing a progressive decline. To avoid damaging spiny cactiforms *Euphorbia* species through stem cutting during sampling, a non-destructive sampling method was previously introduced by Fehlberg et al. (2013) for this type of spiny cacti plant. Following this approach, samples consisting of 5 to 10 pairs of spines were collected from the upper part of the stems of *E. resinifera* individuals in the Foum-El-Anceur region near the city of Beni Mellal, Morocco (32°22.1140’N, 6°14.8390’W). The number of spine pairs collected per individual varied according to spine size to ensure sufficient material for DNA extraction. Spines were clipped using sterile eyebrow forceps and immediately placed into paper bags for transport.

The sampled population was considered homogeneous, consisting of semi-spherical bushes with tightly packed stems. A voucher specimen was deposited in the herbarium of the Scientific Institute, Mohammed V University, Rabat, Morocco (voucher number: RAB114831). The complete annotated chloroplast genome sequence has been deposited in NCBI GenBank under accession number PZ168867.

Genomic DNA was extracted from 5 to 10 spines, corresponding to approximately 20 mg of frozen and ground tissue, into a 2 mL microcentrifuge tube. DNA extraction was performed using the DNeasy Plant Mini Kit (Qiagen, Morrisville, USA) following the manufacturer’s instructions. DNA concentration and purity were assessed using a Nanodrop spectrophotometer, and the extracted DNA was stored at -20 °C until sequencing.

### 2.2 DNA sequencing, chloroplast genome assembly, and annotation

DNA libraries were prepared from 50 ng of genomic DNA using the NEBNext Ultra II FS DNA Library Prep Kit (New England Biolabs). The resulting libraries were sequenced on an Illumina NovaSeq 6000 platform (S2 flow cell); to generate 2 × 150 bp paired-end reads. Raw sequencing reads were adapter-trimmed and quality-filtered using Trimmomatic v0.39 (Bolger et al., 2014). Read quality was then assessed using FastQC v0.12.1 (Andrews, 2010).

The chloroplast genome was *de novo* assembled using NOVOPlasty v4.3.5 (Dierckxsens et al., 2020), with the *rbcL* gene of *Euphorbia ampliphylla* used as the seed sequence. Assembly quality was validated using GetOrganelle v1.7.7.0 (Jin et al., 2020). Genome annotation was performed using GeSeq v2.03 (Tillich et al., 2017), CPGAVAS2 (Shi et al., 2019)), and OGDRAW (Greiner et al., 2019).

### 2.3 SSR analysis and IR/SC boundary characterization

Simple sequence repeats (SSRs) were identified using MISA (Beier et al., 2017), with minimum repeats thresholds of 10, 6, 4, 4, 3, and 3 units for mono-, di-, tri-, tetra-, penta-, and hexanucleotides motifs, respectively. The boundaries of the inverted repeat (IR) regions and the junction between the large single-copy (LSC) and small single-copy (SSC) regions were comparatively analyzed for *E. resinifera* and 19 related *Euphorbia* species using IRscope (Díez Menéndez et al., 2023).

### 2.4 Phylogenetic and compositional analyses

#### 2.4.1 SSR-based compositional analysis

Simple sequence repeat motif presence and absence were encoded as a binary matrix (1/0) for ten representative *Euphorbia* species (a subset of the 22 total spanning all four subgenera and major morphological forms). Sørensen dissimilarity indices were calculated and used for hierarchical clustering based on Ward’s minimum variance method (Ward.D2) (Sorensen, 1948; Ward, 1963). Clustering robustness was assessed via Mantel tests (999 permutations) comparing SSR-based and phylogenetic distance matrices. Additionally, T/A ratios and A+T nucleotide composition were computed to quantify compositional divergence among four *Euphorbia* species within section *Euphorbia.*

#### 2.4.2 Chloroplast genome-based phylogenetic analysis

Chloroplast genome sequences from 22 *Euphorbia* species (including *E. resinifera*) and three Euphorbiaceae outgroups (*Mallotus peltatus, Hevea brasiliensis*, and *Croton tiglium*) were aligned using MAFFT v7.505 (Katoh et al., 2018). Maximum likelihood phylogenies were inferred with IQ-TREE v2.0.7 (Minh et al., 2020) under the GTR+G substitution model with 1,000 bootstrap replicates to assess node support. Resulting trees were visualized using iTOL v6 (Letunic and Bork, 2024) and FigTree v1.4.5 (Rambaut et al., 2018). Genome-wide nucleotide diversity (Pi) was calculated across the 22 *Euphorbia* species using DnaSP v6.12.03 (Rozas et al., 2017) with a sliding window approach (window length = 600 bp, step size = 200 bp) to identify highly variable plastid regions. For *rpl22*- specific analyses, gene sequences were downloaded from NCBI and aligned with MAFFT, with phylogenetic trees constructed using IQ-TREE. Sequence variation, including single-nucleotide polymorphisms, indels, diagnostic trinucleotide motifs, and T/A ratios, was characterized via BLASTN (Altschul et al., 1990) to evaluate its taxonomic discrimination capacity within sect. *Euphorbia*.

## 3 Results

### 3.1 Structural features of the *E. resinifera* chloroplast genome

The chloroplast genome of *Euphorbia resinifera* was assembled from 13,167,418 sequencing reads, of which 6.97% (917,770 reads) corresponded to organelle DNA. A total of 904,376 reads were successfully retained in the final assembly, resulting in an average coverage depth of 849×.

**Figure 1:**
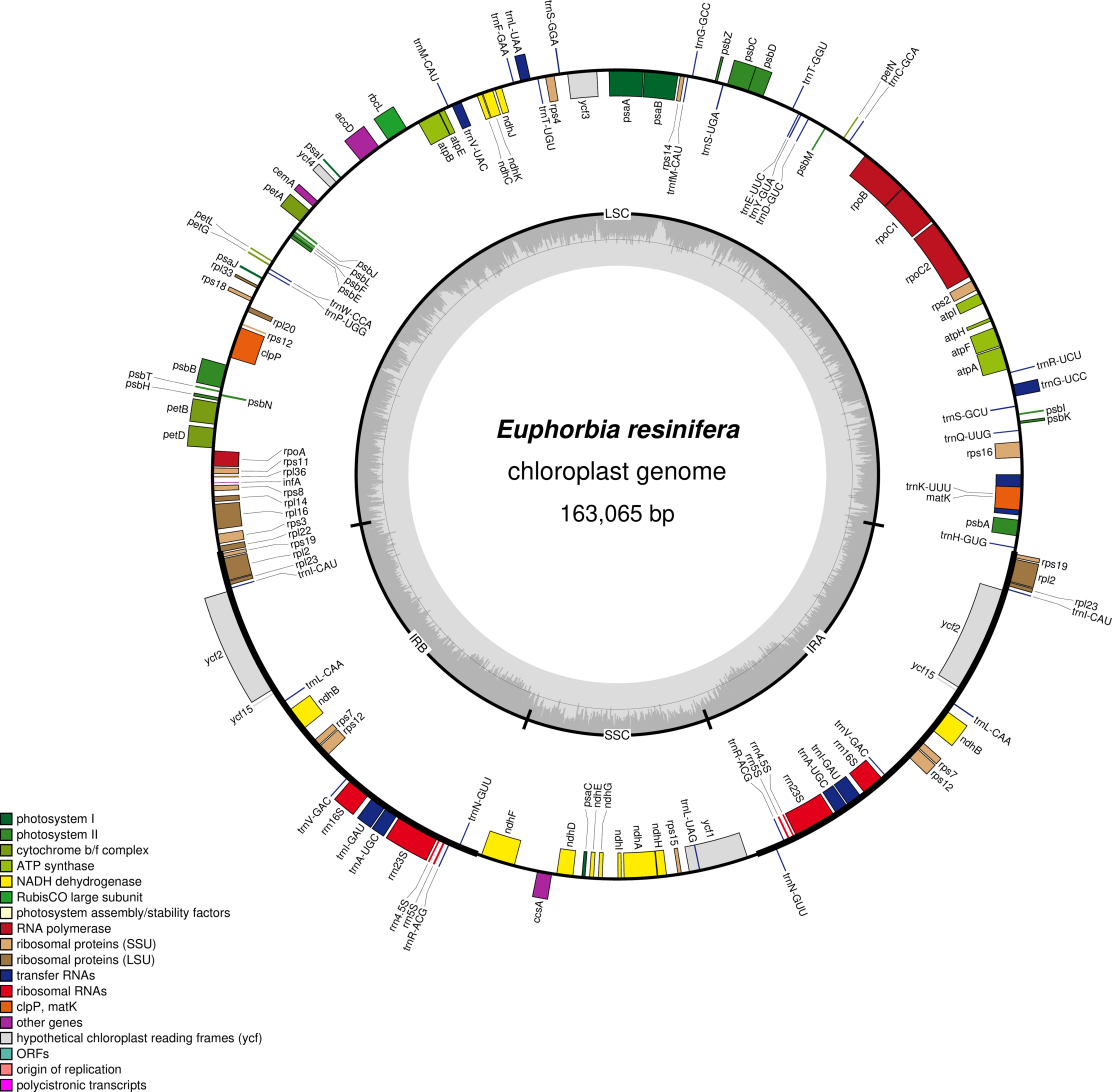
Chloroplast genome map of *E. resinifera*. Genes shown on the outside of the circle are transcribed clockwise, while those on the inside are transcribed counterclockwise. The inner gray track represents GC content variation. The four structural regions (LSC, SSC, and two IRs) are indicated with their corresponding lengths. Genes are color-coded according to functional categories. The center shows typical morphological features of *E. resinifera* and genome statistics (size and GC content).

The complete plastome is 163,065 bp in length with an overall GC content of 35.11% and exhibits the typical quadripartite structure (**Figure 1**), consisting of a large single-copy (LSC) region of 91,462 bp (32.06% GC), a small single-copy (SSC) region of 18,285 bp (29.13% GC), and two inverted repeats (IRs) of 26,659 bp each (42.39% GC). The genome encodes a total of 132 annotated genes: 87 protein-coding genes, 37 tRNA genes, and 8 rRNA genes. Gene distribution comprises 82 genes in the LSC region, 12 in the SSC region, and 19 genes in each IR region. Eight protein-coding genes are duplicated in the IR regions (*ndhB, rpl2, rpl23, rps7, rps12, rps19, ycf2, ycf15*), along with 7 tRNA genes and 4 rRNA genes. (**Tables 1**, **2**).

**Table 1:** Characteristics of the complete chloroplast genome of *Euphorbia resinifera*.

Category	Items	Characteristics
Construction of chloroplast genome	LSC region (bp)	91,462
	SSC region (bp)	18,285
	IRa region (bp)	26,659
	IRb region (bp)	26,659
	Size of chloroplast genome (bp)	163,065
Gene content	Protein-coding genes	87
	tRNA	37
	rRNA	8
	Duplicate genes	19
	Genes on LSC region	82
	Genes on SSC region	12
	Genes on IRa region	19
	Genes on IRb region	19
	Total genes	132
GC content	GC content of LSC region (%)	32.06
	GC content of SSC region (%)	29.13
	GC content of IRa region (%)	42.39
	GC content of IRb region (%)	42.39
	Overall GC content (%)	35.11

**Table 2:** Genes annotated in the chloroplast genome of *Euphorbia resinifera*.

Category of genes	Group of genes	Gene name
Photosynthesis-related Genes	Photosystem II	*psbA, psbB, psbC,psbD, psbE, psbF, psbH, psbI, psbJ,psbK, psbL, psbM,psbN, psbT, psbZ*
	Large subunit of rubisco	*rbcL*
	Photosystem I	*psaA, psaB, psaC, psaI, psaJ*
	NADH dehydrogenase	ndhA*, ndhB*, ndhC, ndhD, ndhE, ndhF, ndhG, ndhH, ndhI, ndhJ, ndhK
	Cytochrome b6/f complex	*petA, petB, petD, petG, petL, petN*
	ATP synthase	atpA, atpB, atpE, atpF*, atpH, atpI
Transcription and translation related genes	Small subunit of ribosomal proteins	rps2, rps3, rps4, **rps7**, rps8, rps11, rps12*, *rps14, rps15, rps18, rps19*
	Large subunit of ribosomal proteins	rpl2*, rpl14, rpl16, rpl20, rpl22, **rpl23**, rpl33, rpl36
	RNA polymerase subunits/transcription RNA	rpoA, rpoB, rpoC1*, rpoC2
	polymerase-associated factor	pafI*
RNA genes	Ribosomal RNA	***rrn4.5, rrn5, rrn16, rrn23***
	Transfer RNA	TrnA-UGC*, trnC-GCA*, trnD-GUC, trnE-UUC, trnF-GAA, trnfM-CAU, trnG-GCC,trnG-UCC*, trnH-GUG,trnI-CAU, trnI-GAU*, trnK-UUU*, **trnL-CAA**, trnL-UAA*, trnL-UAG, trnM-CAU, **trnN-GUU**, trnP-UGG, trnQ-UUG, **trnR-ACG**, trnR-UCU, trnS-CGA, trnS-GCU, trnS-UGA, trnT-GGU, trnT-UGU, **trnV-GAC**, trnV-UAC*, trnW-CCA, trnY-GUA
Other genes	Maturase	*matK*
	Protease	clpP*
	Acetyl-CoA carboxylase	*accD*
	Envelope membrane protein	*cemA*
	Cytochrome c synthesis	*ccsA*
Unknown function		**ycf2**, ycf3*, **ycf15**
Pseudogenes		InfA, rps16*, ycf1

The genes in bold are duplicated, the asterisk indicates genes with introns.

### 3.2 Simple sequence repeats analysis

#### 3.2.1 Analysis of SSR characteristics and distribution in the *E. resinifera* plastome

**Figure 2:**
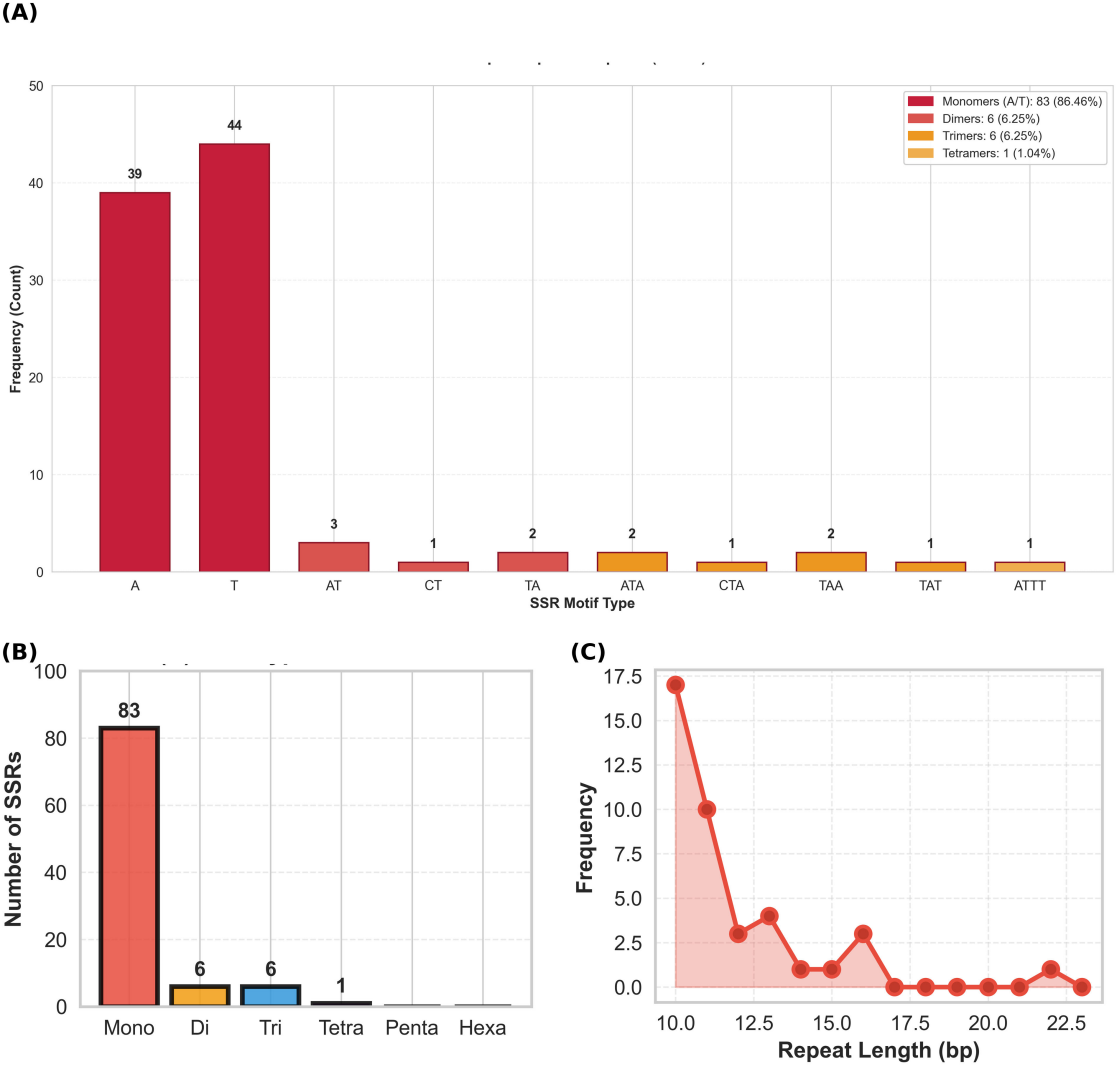
Simple sequence repeat analysis in *Euphorbia resinifera* chloroplast genome: **(A)** SSR type distribution, **(B)** marker classification, **(C)** mononucleotide length distribution.

A comprehensive analysis of the *E. resinifera* chloroplast genome detected 96 SSRs with a high density of 0.59 SSRs/kb. Mononucleotide repeats predominated (83 SSRs, 86.5%), showing a strong bias toward A/T homopolymers (A = 39, T = 44) (**Figure 2A**). Dinucleotide and trinucleotide repeats were equally represented (6 each, 6.2%), whereas only a single tetranucleotide repeats was detected (1.04%) (**Figure 2B**). The length distribution of mononucleotide SSRs exhibited a characteristic exponential decay, with a peak frequency of 17.4 at 10 bp, decreasing to 10.0 at 11 bp and rapidly declining rapidly to near zero beyond 15 bp (**Figure 2C**). This pattern confirms the evolutionary preference for shorter, more stable repeat motifs within chloroplast genomes.

**Figure 3:**
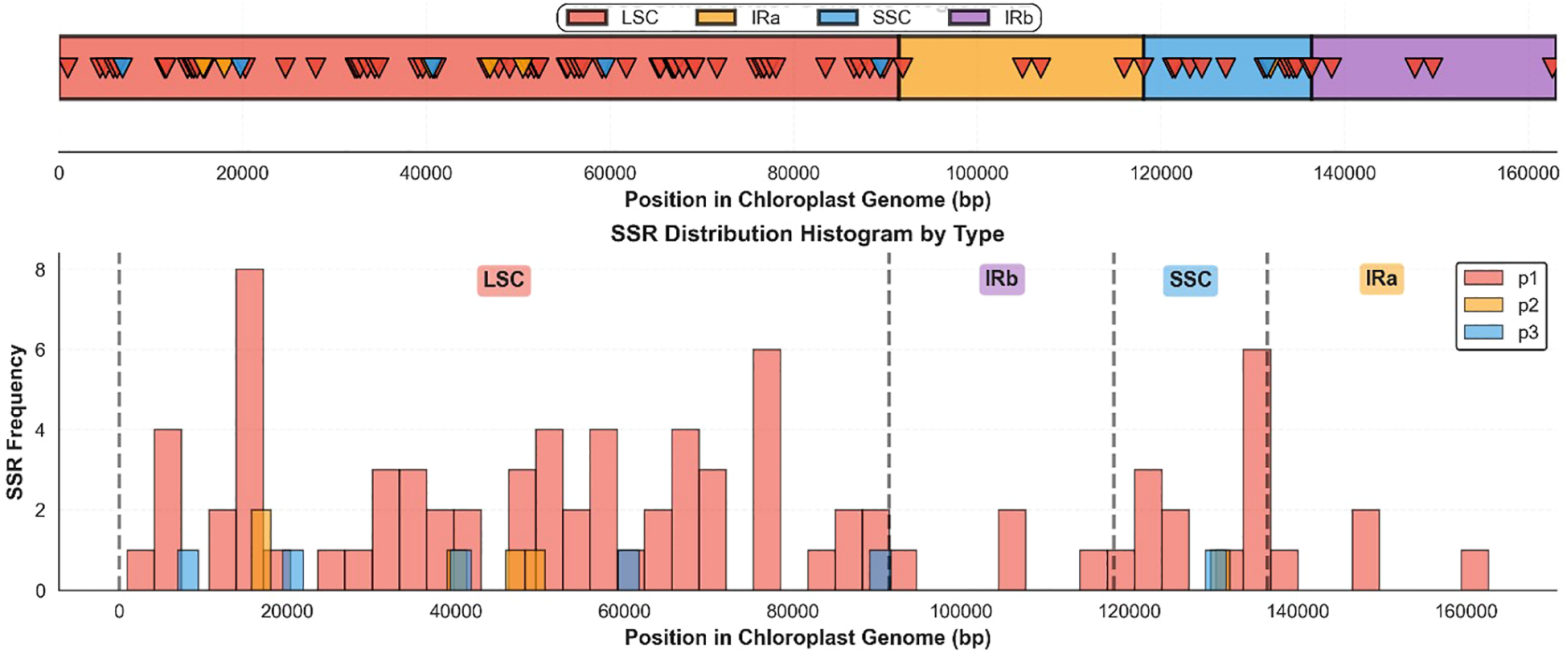
Distribution and density of simple sequence repeats (Mononucleotide (p1), Dinucleotide (p2), and Trinucleotide (p3), across the *Euphorbia resinifera* chloroplast genome regions.

Single-copy regions harbored the majority of SSRs, with the LSC region containing 73 SSRs at the highest density (79.81 per 100 kb), followed by the SSC region with 13 SSRs (71.10 SSRs per 100 kb). In contrast, the IR regions show remarkably low SSR content, with only five SSRs in each IR (18.75 SSRs per 100 kb). Across all genomic regions, mononucleotide repeats (p1) dominate all regions with 83 occurrences, followed by dinucleotide (p2) and trinucleotide (p3) repeats with 6 occurrences each. The single tetranucleotide repeat (p4) was exclusively located within the LSC region (**Figure 3**; **Table 3**).

**Table 3:** Quantitative analysis of SSR distribution across the quadripartite chloroplast genome structure of *Euphorbia resinifera*.

Region	Size (bp)	Number of SSRs	%	p1	p2	p3	p4	Density/100kb
LSC	91,462	73	76.04	62	5	5	1	79.81
SSC	18,285	13	13.54	11	1	1	0	71.10
IRa	26,659	5	5.208	5	0	0	0	18.75
IRb	26,659	5	5.208	5	0	0	0	18.75

#### 3.2.2 Comparative analysis of SSRs across ten *Euphorbia* species: SSR compositional patterns and morphological differentiation

**Figure 4:**
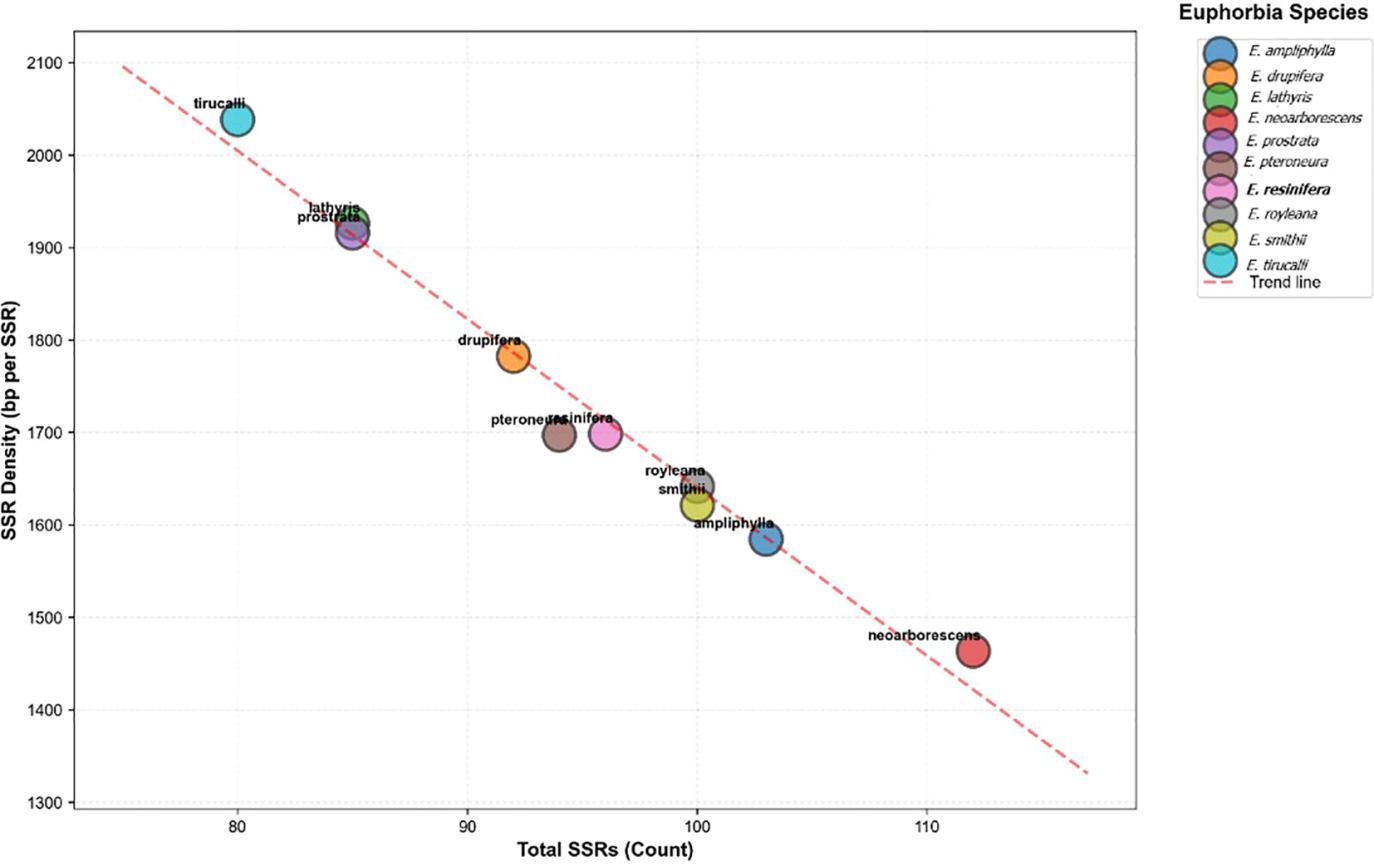
Strong linear correlation (*r* = -0.99) between total SSR number and density across chloroplast genomes of ten *Euphorbia* species.

To assess the evolutionary and compositional significance of SSR patterns in *E. resinifera*, we examined chloroplast SSR characteristics across ten *Euphorbia* species to evaluate genus-wide trends and species-specific variations. *E. resinifera* occupies a central position on the total SSR density regression line (*r* = -0.99; **Figure 4**), showing an intermediate SSR abundance consistent with other cactiform species in the sect. *Euphorbia*.

**Figure 5:**
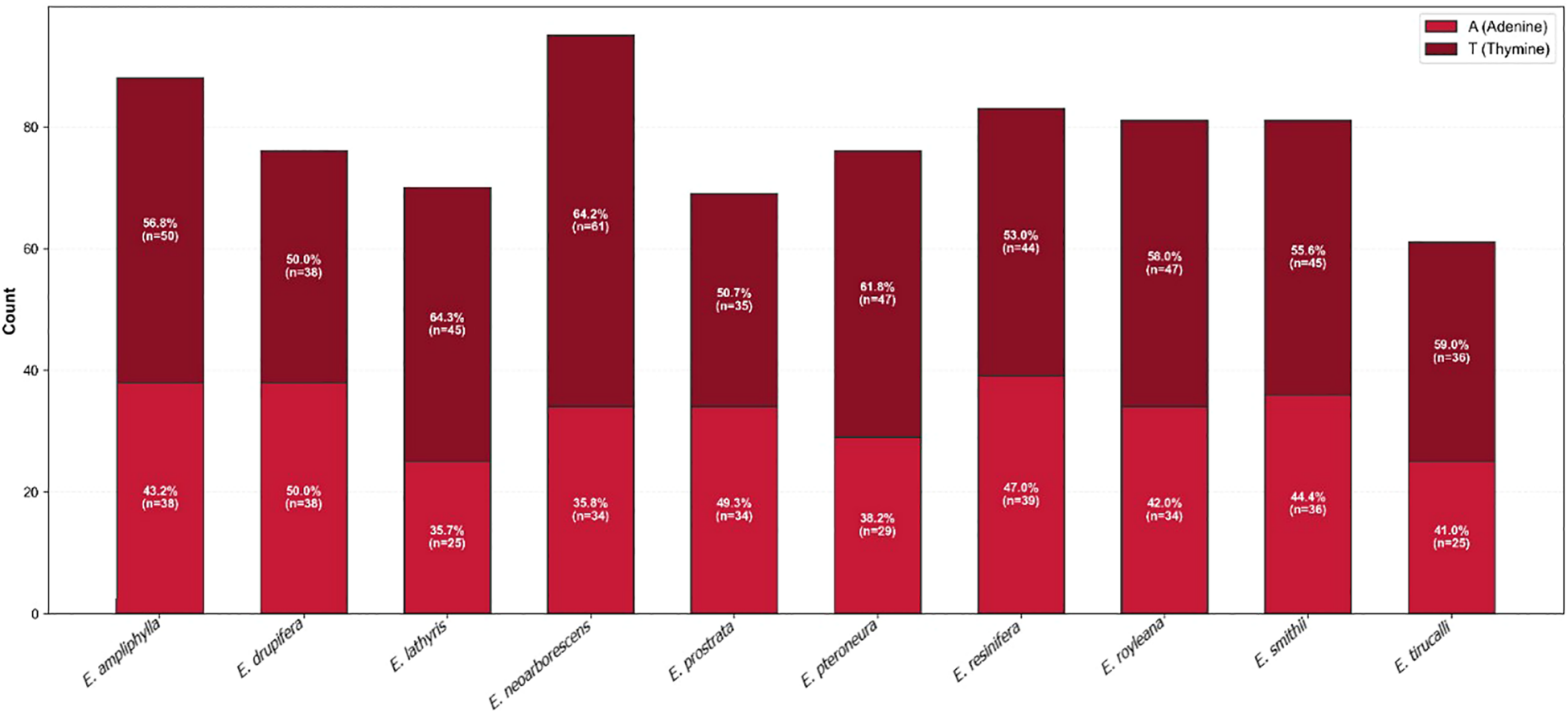
Comparative analysis of A-T, adenine-thymine mononucleotide SSRs distributions in *Euphorbia resinifera* and nine related *Euphorbia* species.

Comparative analysis of adenine-type (A) and thymine-type (T) mononucleotide SSR distribution across complete chloroplast genomes (**Figure 5**) revealed distinct, species-specific compositional patterns within the genus. The pachycaul species *E. drupifera* uniquely exhibits perfect A=T equilibrium (50% each), while the annual herb *E. prostrata* shows near-equilibrium with a slight T enrichment 50.7%. In contrast, most congeners display a pronounced T-enrichment, particularly *E. neoarborescens*, which exhibits the highest SSR abundance with strong T enrichment (64.2% T), and *E. lathyris* (64.3% T). *E. resinifera* shows a relatively balanced AT composition (47% A, 53% T), consistent with its intermediate SSR profile and providing a distinctive compositional signature within cactiform *Euphorbia.*

Analysis of the repetition patterns of simple sequence repeat motifs revealed multiple distinct motif classes according to their size, including monomers (A, T), dinucleotides (AT, CT, TA), trinucleotides (ATA, CTA, TAA, TAT, AAT, TTA, ATT, CAA), tetranucleotides (ATTT), as well as pentanucleotide and hexanucleotide motifs (**Supplementary Figure 1**). Sørensen-Dice analysis identified TTA as the most phylogenetically informative motif (index = 0.5, present in 5/10 species), followed by CTA, TAA, AAT, and TATAT (index = 0.3) (**Table 4**). The predominance of A/T-rich motifs reflects the characteristic composition of chloroplast genomes.

**Table 4:** Top phylogenetically informative SSR motifs.

Motif	Present in species	Absent in species	Sørensen-dice index
TTA	5	5	0.5
CTA	3	7	0.3
TAA	7	3	0.3
AAT	7	3	0.3
TATAT	3	7	0.3
ATTT	2	8	0.2
ATATAC	2	8	0.2
AAATAG	2	8	0.2
ATA	9	1	0.1
TAT	9	1	0.1
AAAGA	1	9	0.1
TTATA	1	9	0.1
TGTATA	1	9	0.1
TATAG	1	9	0.1
TAAAA	1	9	0.1

**Figure 6:**
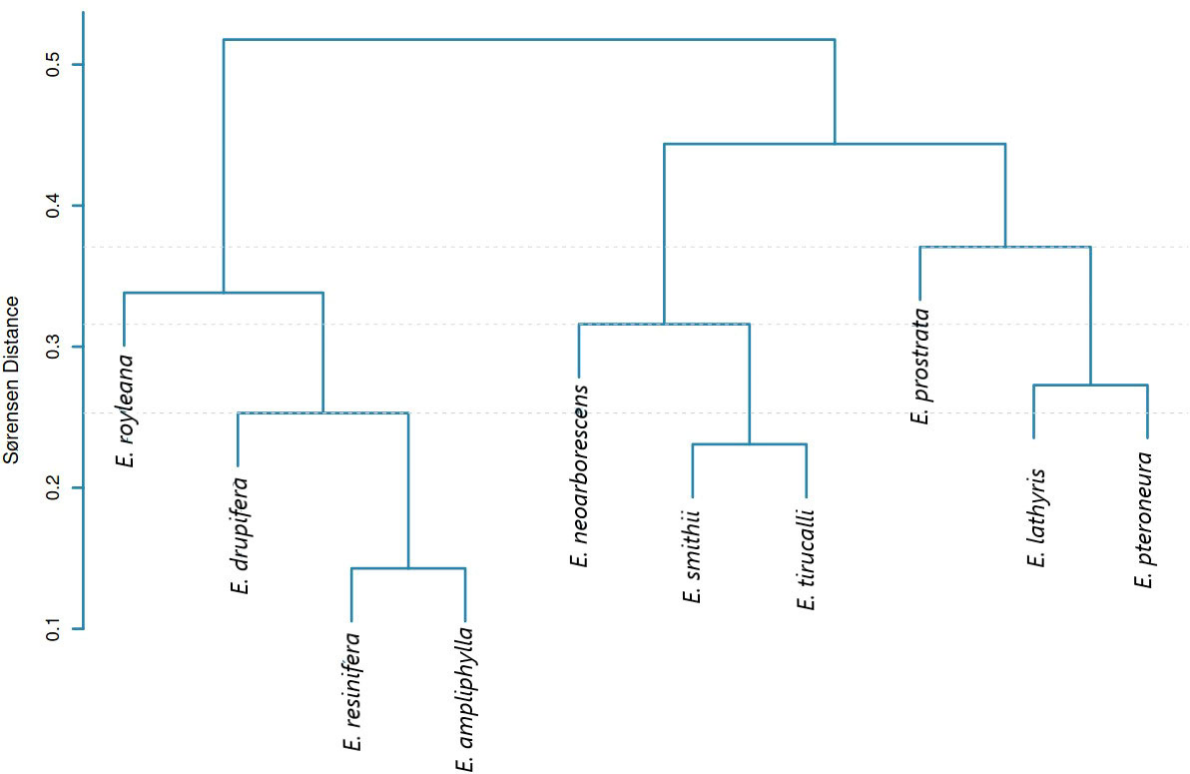
Sørensen distance-based dendrogram showing SSR compositional clustering with taxonomic signal limited to sectional monophyly in sect. *Euphorbia*, while subgeneric relationships are confounded by homoplasy.

The Sørensen dissimilarity matrix reveals three SSR-based compositional clusters with low within cluster dissimilarities (0.14–0.45) and higher between-cluster distances (0.33–0.47). Cluster I groups all four species in sect. *Euphorbia* (*E. resinifera*, *E. ampliphylla*, *E. drupifera*, and *E. royleana*), with *E. resinifera* and *E. ampliphylla* showing highest similarity (0.14) (**Figure 6**; **Supplementary Table 1**). However, Clusters II and III mix species across subgenera: Cluster II associates *E. neoarborescens* and *E. tirucalli* (subgenus *Euphorbia*) with *E. smithii* (subgenus *Athymalus*), while Cluster III groups *E. prostrata* (subgenus *Chamaesyce*), *E. lathyris* (subgenus *Esula*), and *E. pteroneura* (subgenus *Euphorbia*). Thus, cpSSR compositional profiles provide taxonomically informative characters exclusively for resolving sectional monophyly in sect. *Euphorbia*, while lacking phylogenetic signal for higher-order taxonomic relationships due to extensive character state convergence and evolutionary homoplasy.

**Figure 7:**
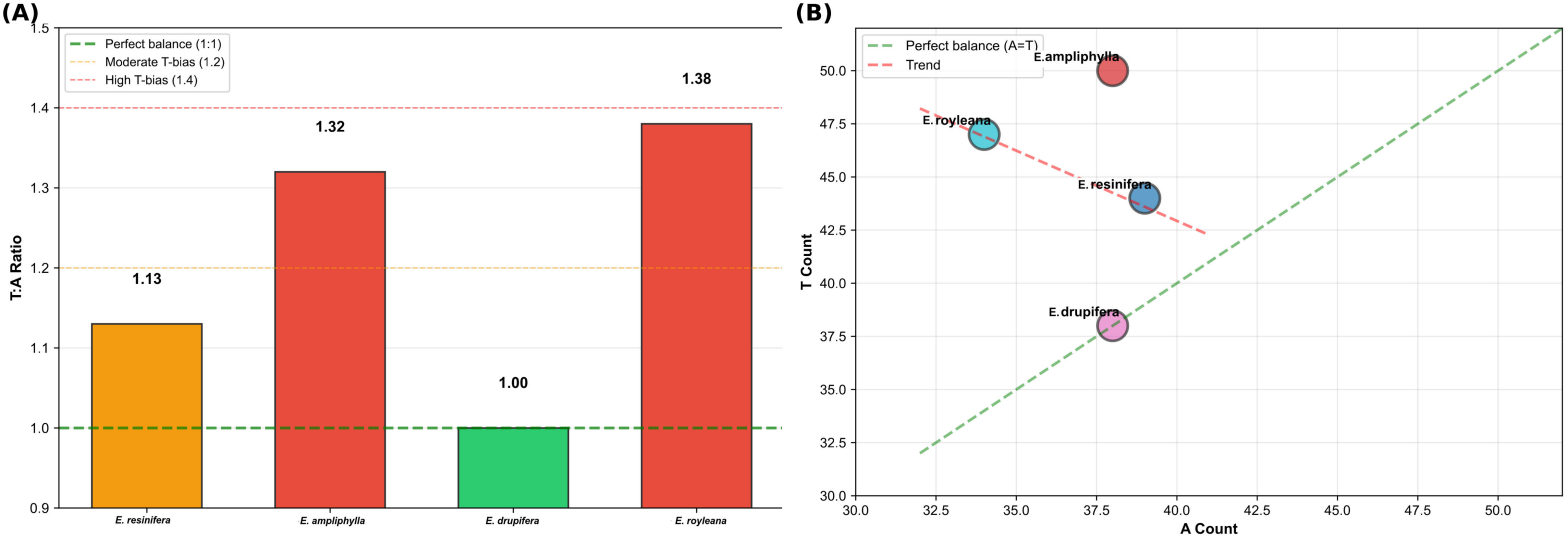
Comparative differentiation of chloroplast SSR monomers among four *Euphorbia* species (sect. *Euphorbia*). **(A)** T/A ratio gradient showing progressive thymine enrichment from pachycaul to cactiform species. **(B)** A-T scatter plot highlighting compositional divergence and revealing molecular basis for taxonomic discrimination between cactiform and pachycaul lineage.

Analysis of the T/A ratio gradient shows gradual enrichment in T bases, progressing from the balanced pachycaul species *E. drupifera* (T/A = 1.00) through increasingly T-enriched cactiform species: *E. resinifera* (1.13), *E. ampliphylla* (1.32) and *E. royleana* (1.38) (**Figure 7**).

### 3.3 Phylogenetic relationships and molecular resolution in sect. *Euphorbia*

#### 3.3.1 Maximum likelihood phylogenetic reconstruction

**Figure 8:**
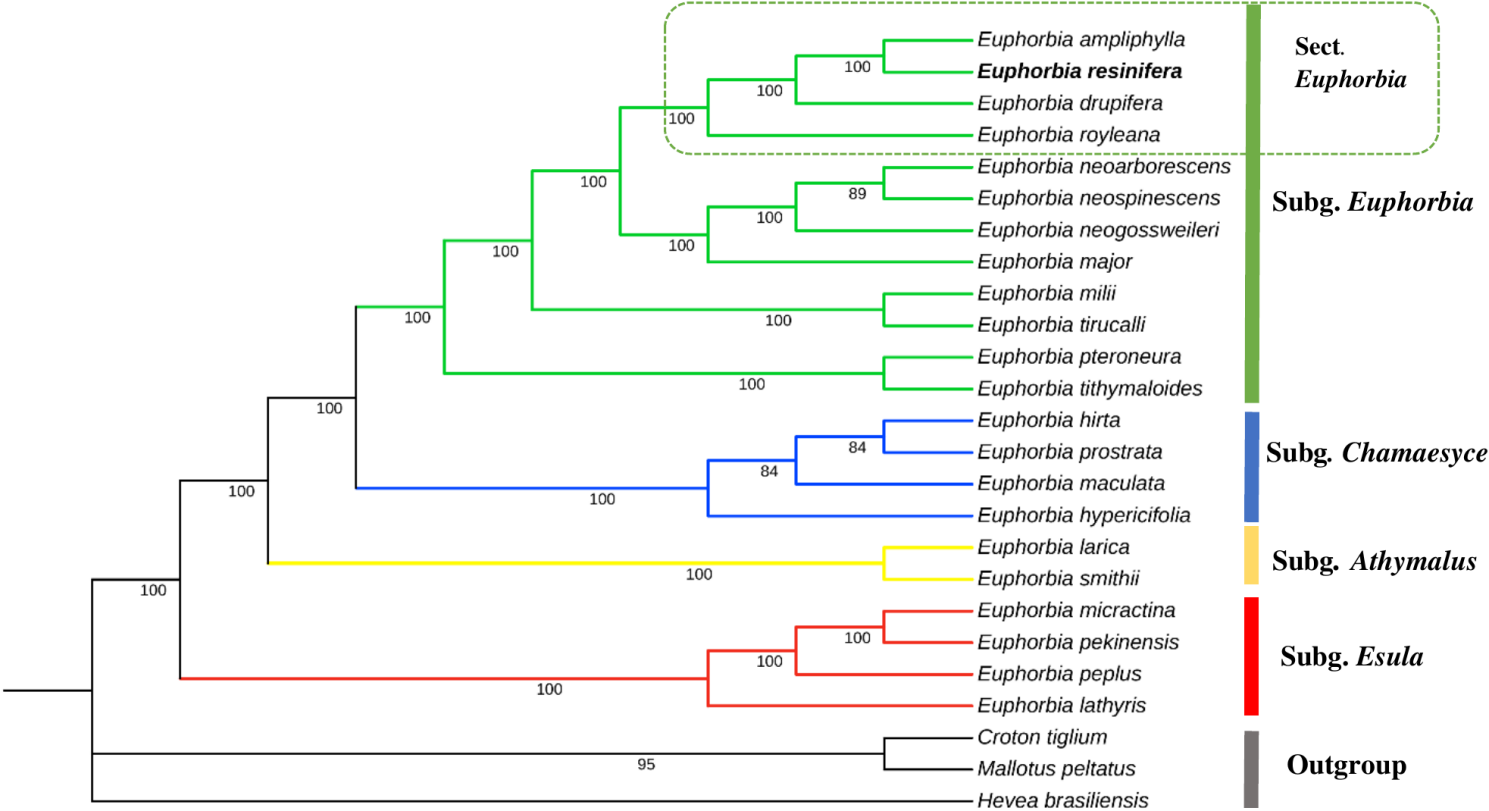
Maximum likelihood (ML) tree based on the chloroplast sequences of 25 species, including *22 Euphorbia* species and three Euphorbiaceae outgroups (*Hevea brasiliensis*, *Croton tiglium*, *Mallotus peltatus*). The sequences incorporated in the tree includes *E. resinifera* (this study) and 24 publicly available chloroplast genomes.

The maximum likelihood (ML) phylogenetic tree shows that *E. resinifera* clusters with other *Euphorbia* species, forming a monophyletic group with maximal bootstrap support (BP = 100). *E. resinifera* is particularly closely related to *E. ampliphylla, E. drupifera*, and *E royleana*, all of which share a common ancestor within sect. *Euphorbia*. However, *E. drupifera* is a non-cactiform species, unlike *E. resinifera, E. ampliphylla* and *E. royleana*, which are xerophytic cactiform species. It is also noteworthy that *E. resinifera, E. ampliphylla* and *E. drupifera* are distributed in Africa, whereas *E. royleana* is native to Asia (**Figure 8**). Overall, these results further suggest a close evolutionary relationship among the 22 *Euphorbia* species, which cluster together on a strongly supported branch (BP = 100%). Moreover, the maximal bootstrap support for the *E. resinifera* and *E. ampliphylla* clade indicates strong confidence in their inferred relationship.

#### 3.3.2 IR/SC boundary analysis in *E. resinifera* and related species

**Figure 9:**
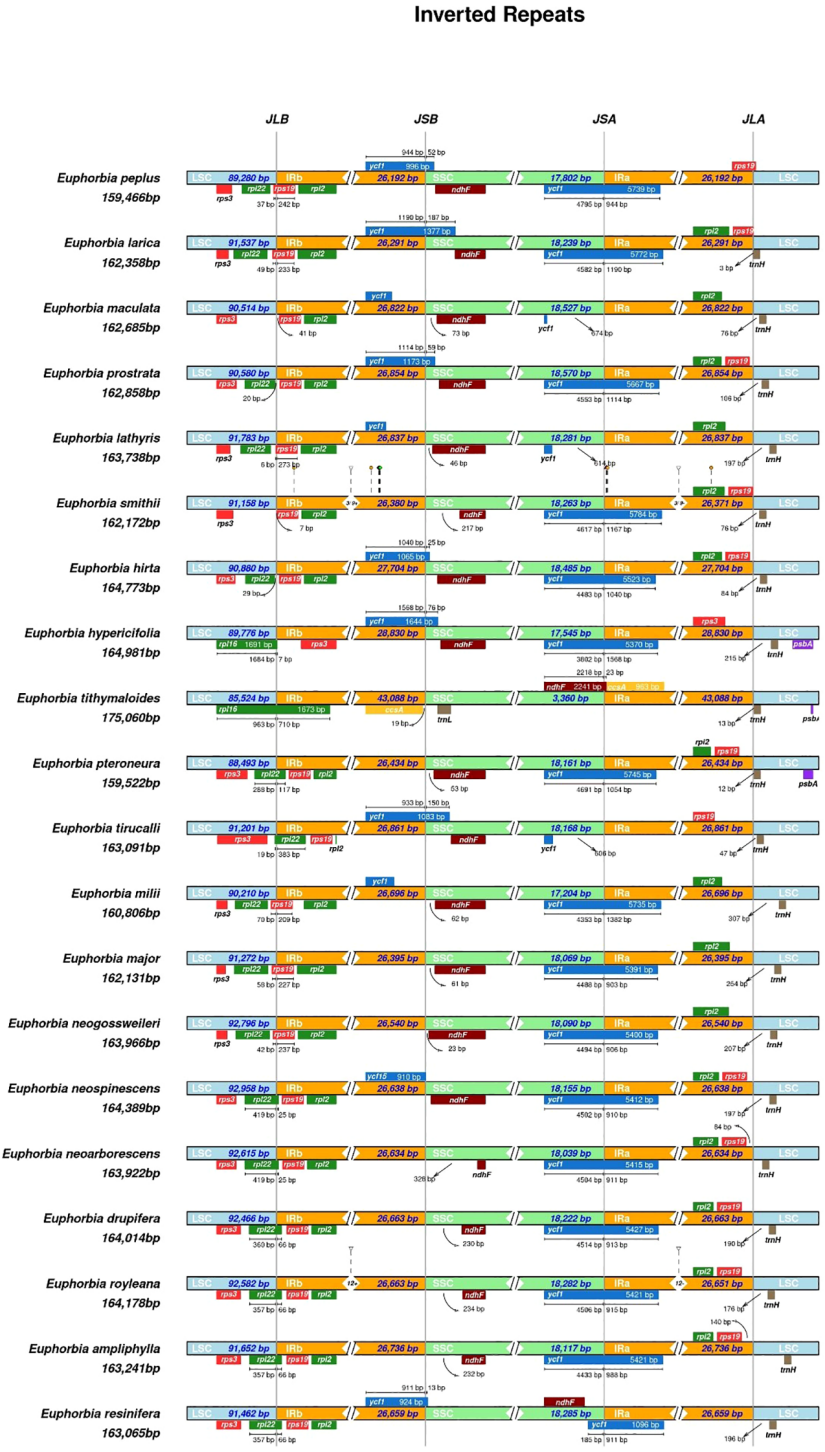
Comparative analysis of IR, inverted repeat and SC, single-copy boundary regions across the chloroplast genomes of *Euphorbia resinifera* and 19 congeneric species within the genus *Euphorbia*.

Comparative analysis of IR/SC boundary regions across 20 *Euphorbia* species revealed substantial interspecific variation in the positioning, orientation, and extension of key genes (*rps19, rpl22, rpl2, trnH, ycf1, and ndhF* (Pi = 0.0091)) at the four junctions (LSC/IRb, IRb/SSC, SSC/IRa, IRa/LSC). Within section *Euphorbia* (*E. resinifera, E. ampliphylla, E. drupifera*, and *E. royleana*), differences were minimal with junction shifts of only 74 bp in the IR, 155 bp in the SSC, and 1,120 bp in the LSC. Among these regions, the *rpl22*/*rps19* boundary at the LSC/IRb junction exhibited the highest variability. *E. resinifera, E. ampliphylla*, and *E. royleana* shared identical *rpl22* positioning (357 bp in the LSC and 66 bp in the IRb), whereas *E. drupifera* showed a slight expansion (360 bp in the LSC, 66 bp in the IRb). Despite these minor differences in the LSC portion, *rpl22* consistently extended 66 bp into the IRb region across all four species within section *Euphorbia*, indicating a conserved structural feature characteristic of this taxonomic group (**Figure 9**).

#### 3.3.3 Nucleotide polymorphism distribution identifies informative markers

**Figure 10:**
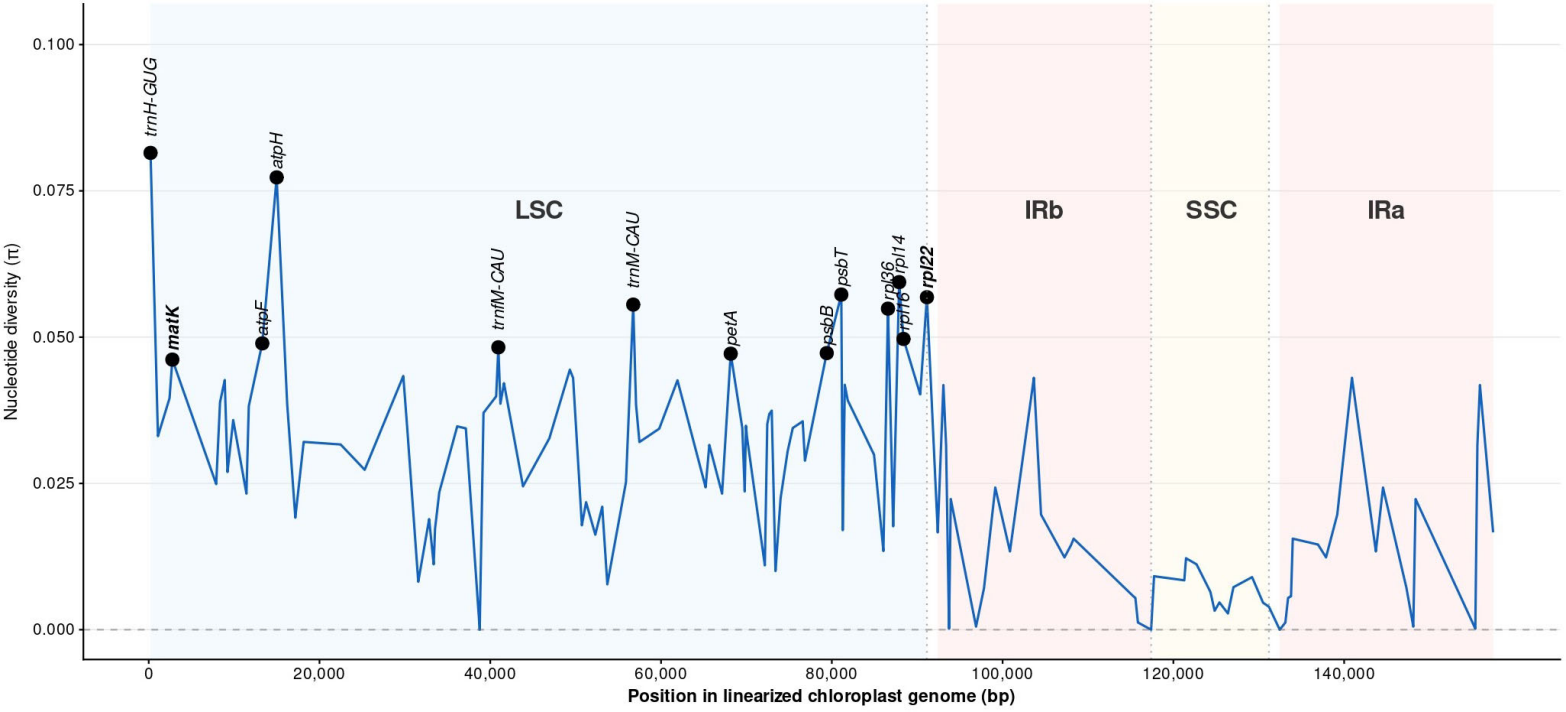
Nucleotide diversity (Pi) across chloroplast genomes of 22 *Euphorbia* species by sliding window analysis, highlighting the top 13 most variable genes.

Comparative nucleotide diversity analysis of 129 chloroplast genes across 22 *Euphorbia* species representing four subgenera reveals uneven distribution of hypervariable regions (HVRs) (**Figure 10**; **Table 5**). The top 13 most variable genes are exclusively LSC-encoded with Pi values ranging from 0.081 (*trnH-*GUG) to 0.046 (*matK*), while 32 highly conserved genes exhibit minimal polymorphism (Pi ≤ 0.012), including *ndhF* (Pi = 0.009), *trnL-*UAG (Pi = 0.008), and *trnL-*CAA (Pi = 0.007), with three tRNA loci showing complete conservation (Pi = 0.000). Notably, *rpl22* (Pi = 0.057, rank 5) exhibits 23% higher nucleotide diversity than traditional markers *matK* (Pi = 0.046), *rbcL* (Pi = 0.034), and *ndhF* (Pi = 0.0091) (**Supplementary Table 3**).

**Table 5:** Distribution of variable genes across chloroplast genome regions in 22 *Euphorbia* species.

Category (pi range)	LSC	SSC	IR	Total	% of total
Highly variable (0.038 - 0.081)	29	0	4	33	25.6%
Variable (0.024 - 0.037)	30	0	2	32	24.8%
Conserved (0.012 - 0.024)	16	0	16	32	24.8%
Highly conserved (0.000 - 0.012)	6	12	14	32	24.8%
Total	81	12	36	129	100%

Regional distribution analysis reveals marked variation across chloroplast genome compartments. The LSC region shows the highest evolutionary rate with 59 of 81 genes (72.8%) classified as variable to highly variable, while Inverted Repeats exhibit strong conservation with only 3 of 18 genes (16.7%) showing variability in each IR copy, and the SSC region contains 12 entirely conserved genes (0.0% variability). Overall, 65 of 129 chloroplast genes (50.4%) exhibited variability (Pi ≥ 0.024).

#### 3.3.4 *rpl22*-based phylogenetic resolution of morphological groups

**Figure 11:**
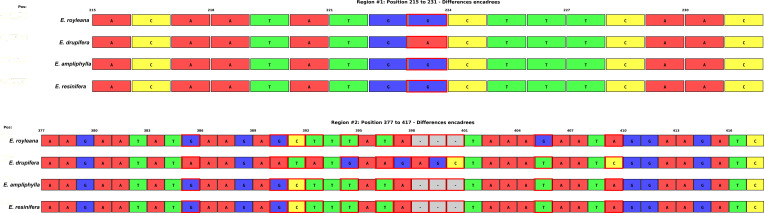
Multiple sequence alignment of the 426-bp *rpl22* gene showing 10 variable sites and a 3-bp indel distinguishing lineages within section *Euphorbia*.

Analysis of the *rpl22* gene reveals two distinct groups: *E. ampliphylla* and *E. resinifera* exhibit 100% sequence identity and share 99.76% similarity with *E. royleana*, while *E. drupifera* displays 2.6% variation (97.41-97.87% similarity) characterized by 10 SNPs and retention of a unique AGC trinucleotide at positions 395–397 that is deleted in the other three species. Nine SNPs (positions 223, 385, 390, 391, 392, 394, 396, 397, 409) are identical across the three cactiform species (*E. resinifera, E. ampliphylla, E. royleana*), indicating a single divergence event from the pachycaul *E. drupifera.* Position 405 represents a biogeographical marker distinguishing Asian *E. royleana* (G) from African taxa (T), confirming biogeographical structure within the otherwise highly conserved gene (96.95% overall identity) (**Figure 11**; **Supplementary Figure 3**). Six mutations align with T/A enrichment through adenine loss (A→G at positions 223, 385, 390; A→T at positions 392, 396) and thymine gain (G→T at position 394), with A→T transversions showing the strongest T/A enrichment effect. Transitions and transversions contribute equally (5 mutations each) (**Table 6**).

**Table 6:** Single nucleotide polymorphisms (SNPs) in the rpl22 gene among four section *Euphorbia* species: comparative analysis of three cactiform and one pachycaul morphotype.

Position	Type	Nucleotide change	*E. drupifera*	*E. resinifera*	*E. ampliphylla*	*E. royleana*	T/A effect
223	Transition	A→G	A	G	G	G	Loses A
385	Transition	A→G	A	G	G	G	Loses A
390	Transition	A→G	A	G	G	G	Loses A
391	Transition	T→C	T	C	C	C	Loses T
397	Transition	G→A	G	A	A	A	Gains A
392	Transversion	A→T	A	T	T	T	Loses A, Gains T
394	Transversion	G→T	G	T	T	T	Gains T
396	Transversion	A→T	A	T	T	T	Loses A, Gains T
409	Transversion	C→A	C	A	A	A	Gains A
405	Transversion	T→G	T	T	T	G	Loses T

**Figure 12:**
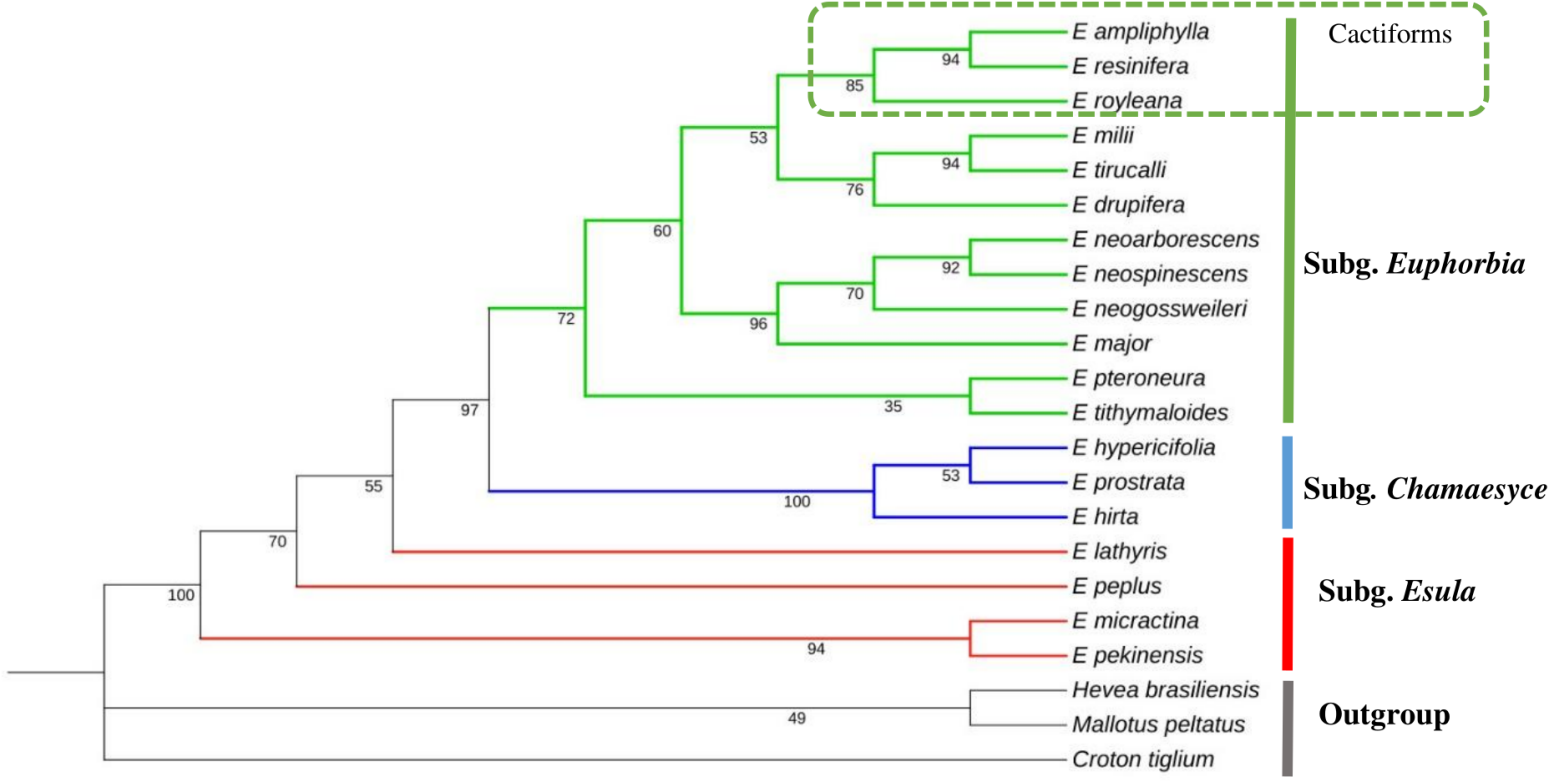
Maximum likelihood *rpl22* phylogeny of 19 *Euphorbia* species with Euphorbiaceae outgroups resolving three major subgenera with high bootstrap support: *Euphorbia* (green, BS = 97%), *Chamaesyce* (blue, BS = 100%), and *Esula* (red, BS = 70%). *E. drupifera* is excluded from sect. *Euphorbia* cactiform clade (*E. ampliphylla, E. resinifera, E. royleana*; BS = 85%).

Phylogenetic analysis of the *rpl22* gene (306–537 bp) from 22 species confirms the monophyly of *Euphorbia* with maximum bootstrap support (BS = 100%), covering the subgenus *Euphorbia* (green, BS = 97%), the subgenus *Chamaesyce* (blue, BS = 100%) and the subgenus *Esula* (red, BS = 70%). Within the subgenus *Euphorbia*, the section *Euphorbia* includes the three cactiforms species *E. ampliphylla, E. resinifera*, and *E. royleana* (BS = 85%), with *E. ampliphylla and E. resinifera* being identical over 423 bp (0 SNPs) and *E. royleana* differing by a single SNP. The *Monadenium* section is well resolved (BS = 96%), with *E. neospinescens* and *E. neoarborescens* showing 100% sequence identity over 444 bp, while the herbaceous *E. neogossweileri* appears as their sister taxon (BS = 70%). The subgenus *Chamaesyce* is the best-supported clade in the tree (BS = 100%). The most notable result concerns *E. drupifera*, whose pachycaul morphology had previously obscured its taxonomic position; the *rpl22* gene excludes it from the sect. *Euphorbia* and resolves its position with *E. milii* and *E. tirucalli* (BS = 76%). The subgenus *Esula* has limited internal resolution, with only *E. micractina* and *E. pekinensis* forming a well-supported clade (BS = 94%), these two species being 100% identical over 306 bp, while the other species appear as isolated branches (**Figure 12**).

## 4 Discussion

The *Euphorbia resinifera* chloroplast genome (163,065 bp, 35.11% GC, 132 genes) exhibits typical quadripartite structure within the ranges reported for *Euphorbia* species by Lee et al. (2024) and Wei et al. (2021), demonstrating remarkable structural conservation across the genus. The low GC content (35.11%) matches other subgenus *Euphorbia* species (*E. drupifera, E. neogoswelleri, E. tithymaloides*) reported by Wei et al. (2021), supporting the phylogenetic coherence of this taxonomic section and providing valuable baseline data for future comparative genomic studies.

Simple sequence repeat analysis revealed strong A/T bias, with mononucleotide repeats comprising 86.5% of all detected SSRs and A/T homopolymers predominating (A = 39, T = 44), consistent with widespread patterns in land plant plastomes where A/T-type SSRs frequently exceed 90% (Kan et al., 2024), reflecting characteristic chloroplast genome composition (Kuang et al., 2011; Asaf et al., 2018; Xu et al., 2025) and attributable to polyadenylation processes and the relative ease of A/T strand separation during replication increasing slippage probability (Dodsworth et al., 2015). The AT-rich regions are often associated with easier unwinding of DNA during transcription and potentially more efficient and accurate translation processes (Wang et al., 1984). The prevalence of these A/T-rich repetitive sequences is thought to contribute to genomic plasticity and stability, with their specific motifs and distribution patterns likely reflecting evolutionary pressures that shaped plant genomes for adaptation and divergence (Wu et al., 2018; Dong et al., 2025).SSR density is approximately four times higher in single-copy regions than in inverted repeats, consistent with substitution rates 3.7 times slower in IRs (Zhu et al., 2016)), while IR boundary contraction and expansion events drive interspecific genome size variation (Shen et al., 2017; Corvalán et al., 2023). While cpSSR compositional profiles successfully recover sect. *Euphorbia* as a distinct cluster, their failure to resolve established subgeneric boundaries in Clusters II and III confirms the well-documented limitations of microsatellite markers for deep phylogenetic inference. Although microsatellite markers are prone to homoplasy (Kuang et al., 2011; Dodsworth et al., 2015; Vieira et al., 2016) this limitation has been addressed by incorporating whole-chloroplast sequencing to directly examine underlying sequence variation (Wheeler et al., 2014).

Complete chloroplast genome analyses confirm the monophyly of the four *Euphorbia* subgenera (*Esula*, *Chamaesyce*, *Athymalus*, and *Euphorbia*) and place *E. resinifera* within section *Euphorbia*, together with *E. ampliphylla*, *E. drupifera*, and *E. royleana*, in agreement with previous taxonomic classifications (Horn et al., 2012; Dorsey et al., 2013; Wei et al., 2021). Within sect. *Euphorbia*, the two African cactiform species (*E. ampliphylla* and *E. resinifera*) form a sister group, and together with the pachycaul *E. drupifera* constitute a well-resolved African clade, with the Asian *E. royleana* occupying a basal position relative to this clade, demonstrating that biogeographic origin strongly influences plastome evolution. However, the grouping of the pachycaul *E. drupifera* with African cactiform species in the complete plastome analysis, despite its fundamentally distinct morphology, illustrates how shared biogeographic origin can override true phylogenetic signal, consistent with the well-documented influence of geography on chloroplast genome evolution (He et al., 2024).

A key architectural distinction characterizes *Euphorbia* species within the section *Euphorbia*: atypical positioning of the *rpl22* gene spanning the LSC/IRb junction (357–360 bp in LSC and 66 bp in IRb), in contrast to its conventional localization entirely within the LSC in most angiosperm plastomes (Meng et al., 2018; Ahmed and Rahman, 2024). Similar boundary variations have been documented in *Paphiopedilum* orchids, where *rpl22* extends into both regions in 6 of 14 species (Guo et al., 2021). This configuration represents a stable synapomorphy among cactiform taxa (*E. resinifera, E. ampliphylla*, and *E. royleana*), whereas the pachycaul species *E. drupifera* exhibits slight boundary modification (360 bp in LSC/66 bp in IRb), indicating a subtle structural divergence. This unique positioning subjects *rpl22* to distinct evolutionary dynamics, potentially influenced by the homogenizing effects of gene conversion in the IR region while maintaining sufficient variation in the LSC portion, consequently offering effective infrageneric resolution by balancing variability and conservation (Nie et al., 2025).

Regional polymorphism analysis highlights the limited suitability of conventional plastid markers for resolving fine-scale phylogenetic relationships in *Euphorbia*. Compartment-specific evolutionary patterns reveal pronounced LSC dominance in sequence variation, while IR regions maintain strong conservation through gene conversion mechanisms, with highly variable regions (HVRs) predominantly concentrated in non-coding sequences, similar to patterns observed across angiosperms (Shi et al., 2023; Xu et al., 2025). This evolutionary heterogeneity reflects fundamental differences in selective constraints, where IR-SC junctions remain highly conserved with stable boundary markers (*rps19, ycf1, ndhF, trnN, trnH*), contrasting sharply with LSC-driven variability.

Despite this compartment-specific variation, widely used phylogenetic markers demonstrate variable and often insufficient discriminatory power for species-level resolution. While some markers like *matK* exhibit high variability, others including *rbcL, psbA, ndhF, trnL*-UAG, and *trnL*-CAA show limited phylogenetic informativeness, with multiple studies demonstrating inadequate resolution for discriminating closely related species in taxonomically complex genera (Muellner et al., 2003; Shi et al., 2023; Nie et al., 2025). This systematic limitation necessitates identification of alternative loci with enhanced phylogenetic utility. In contrast, *rpl22* exhibits exceptional species-level discriminatory utility, aligning with comparative genomics studies identifying *rpl22* and its flanking sequences as evolutionarily dynamic mutation hotspots suitable for DNA barcoding applications (Wang et al., 2021, 2024). The documented positive selection in *Euphorbia altotibetica* (Qu et al., 2025) and *Cistanche* species (Miao et al., 2022) indicates rapid adaptive evolution in this ribosomal protein L22-encoding gene, suggesting functional significance for maintaining protein synthesis and stress adaptation in arid environments. This combination of evolutionary dynamism and potential functional adaptation establishes *rpl22* as a promising phylogenetic marker for systematic studies in xerophytic lineages where traditional plastid markers lack sufficient resolution.

Phylogenetic analysis based on Maximum Likelihood reconstruction demonstrates that the *rpl22* gene better reflects morphological relationships than complete chloroplast genome phylogeny. While whole-plastome analyses cluster species by geographic origin, *rpl22* correctly excludes *E. drupifera* from sect. *Euphorbia* based on molecular grounds consistent with its distinct pachycaul morphology. As noted by Dorsey et al. (2013), although most species of sect. *Euphorbia* possess spiny succulent stems, leafy trees such as *E. drupifera* represent morphological exceptions, with persistent leaves interpreted as ancestral state reversal (Horn et al., 2012).

The 2.6% molecular distinction of *E. drupifera* (*rpl22*) and its T/A ratio (cpSSRs) of 1.00, contrasting with values of 1.13–1.38 in cactiform species, may correlate with fundamental photosynthetic strategy differences between stem-dominated and leaf-dominated growth forms (Horn et al., 2012). Remarkable sequence conservation among morphologically similar species (0–1 SNPs among cactiform species of 423 bp; 100% identity between *E. neospinescens* and *E. neoarborescens* of 444 bp and *E. micractina* and *E. pekinensis* of 306 bp) demonstrates that *rpl22* reflects deep evolutionary relationships rather than recent biogeographic history. These findings highlight the value of targeted single-gene approaches for resolving systematic relationships in hyperdiverse genera where geographic clustering can confound whole-plastome phylogenetic inference.

## 5 Conclusion

This study demonstrates that integrating multiple independent molecular approaches provides robust taxonomic resolution in *Euphorbia*, overcoming individual methodological limitations including microsatellite homoplasy. Using a novel non-destructive spine-DNA genome-skimming protocol, we generated the first complete chloroplast genome of the endemic arid-adapted cactiform *E. resinifera* from North-West African hyperarid zones. Our findings establish *rpl22* as a superior phylogenetic marker that differentiates cactiform xerophytes from pachycaul species through lineage-specific substitution patterns and diagnostic SNPs correlating with photosynthetic architectural transitions, distinctions undetectable using conventional plastid markers (*rbcL, matK, ndhF*). The *rpl22* gene resolves taxonomic boundaries congruent with whole-genome classifications while achieving maximum bootstrap support for *Euphorbia* relationships. Concatenation with additional genes is needed to enhance phylogenetic robustness and remove evolutionary ambiguities in taxonomic relationships.

## Data Availability

Data are deposited in: Zenodo: https://doi.org/10.5281/zenodo.18750510 (gene lists, phylogenetic files, CDS verification, pseudogenes, tRNA/rRNA data). NCBI GenBank: BioProject PRJNA1371610, BioSample SAMN53505053, accession number PZ168867 (BankIt3063561).
