# Supplementary material for: Complete chloroplast genome of Euphorbia resinifera: overcoming biogeographical bias in phylogenetic inference and establishing a conservation genomics framework for threatened North-West African cactiform species
Source: Front Plant Sci. 2026 Apr 1;17:1785579. doi: 10.3389/fpls.2026.1785579 (PMC13086001; doi:10.3389/fpls.2026.1785579)
Supplement: Supplementary file 1 [file DataSheet1.docx]

Supplementary Material

# Supplementary Figures and Tables

##
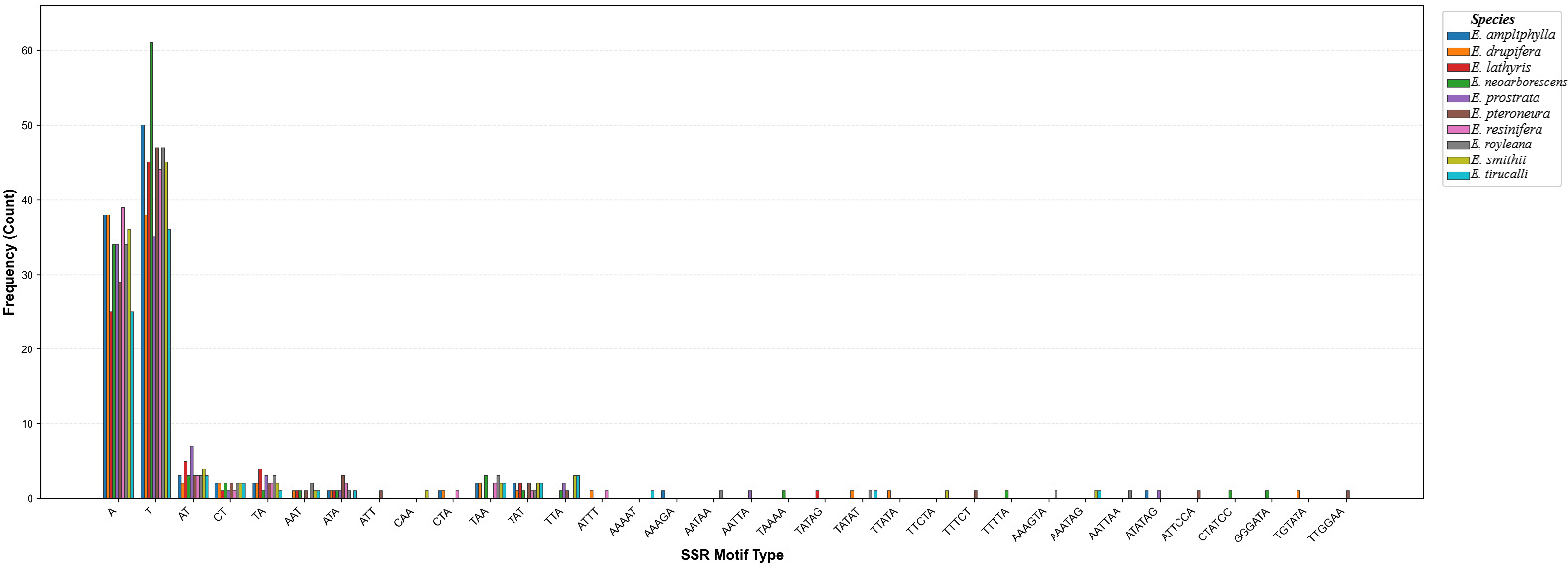
Supplementary Figures

**Supplementary Figure 1.** Comparative SSR motif frequency distribution across 10 *Euphorbia* species including *E. resinifera*


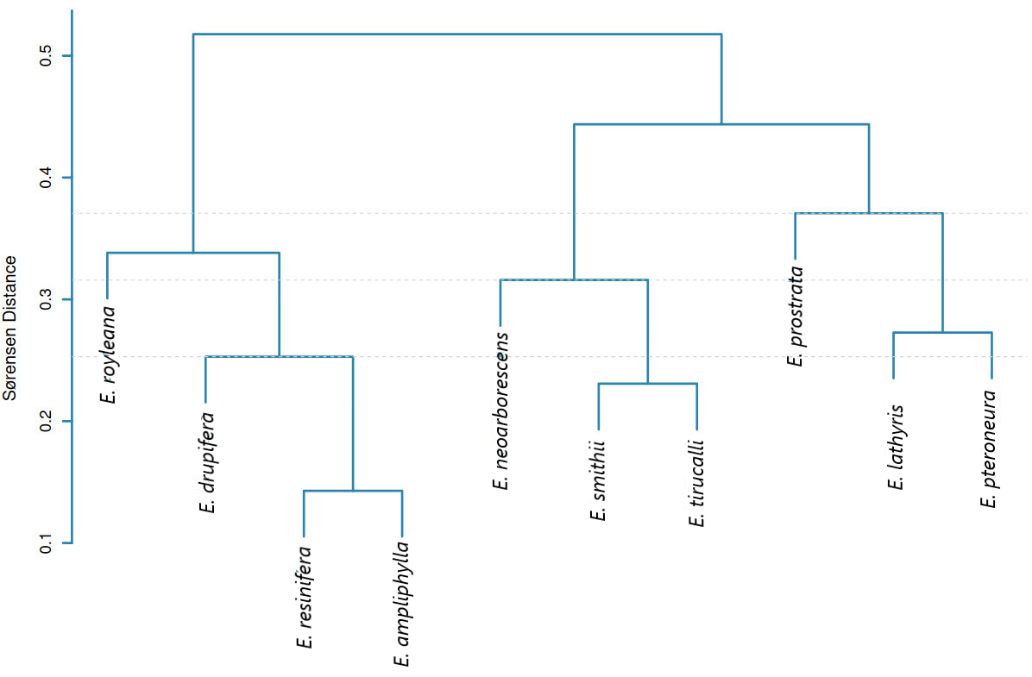


**Supplementary Figure 2.** Dendrogram showing phylogenetic relationships among *Euphorbia* species inferred from SSR markers using the homoplasy-resistant Sørensen distance


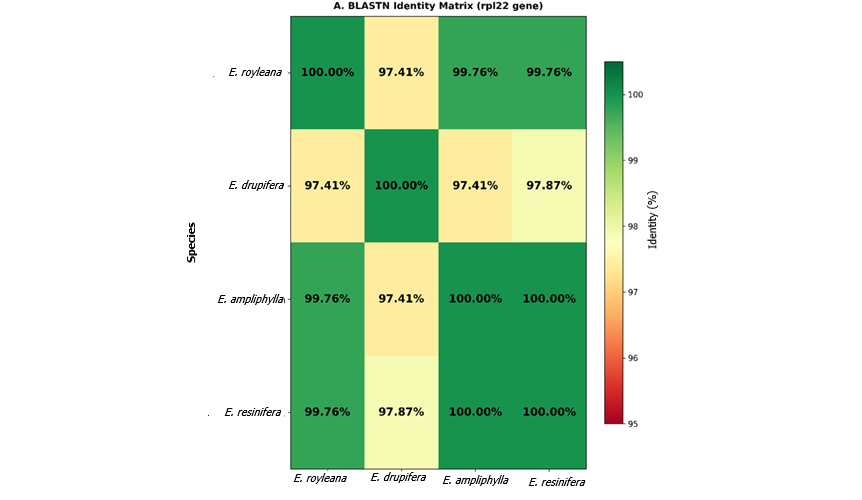


**Supplementary Figure 3.** Sequence similarity matrix of the rpl22 gene sequence similarity matrix revealing phylogenetic divergence in section Euphorbia

## Supplementary Tables

**Supplementary Table 1**. Sorensen-based dissimilarity matrix of ten *Euphorbia* species using phylogenetically informative SSR motif composition from chloroplast genome (cpSSRs)

| Species | 1 | 2 | 3 | 4 | 5 | 6 | 7 | 8 | 9 | 10 |
| --- | --- | --- | --- | --- | --- | --- | --- | --- | --- | --- |
| *E. resinifera* | 0.0000000 |  |  |  |  |  |  |  |  |  |
| *E. ampliphylla* | 0.1428571 | 0.0000000 |  |  |  |  |  |  |  |  |
| *E. drupifera* | 0.1666667 | 0.2800000 | 0.0000000 |  |  |  |  |  |  |  |
| *E. lathyris* | 0.2631579 | 0.3000000 | 0.3043478 | 0.0000000 |  |  |  |  |  |  |
| *E.neoborascescens* | 0.3333333 | 0.3600000 | 0.3571429 | 0.3043478 | 0.0000000 |  |  |  |  |  |
| *E. prostrata* | 0.3684211 | 0.3000000 | 0.4782609 | 0.3333333 | 0.3913043 | 0.0000000 |  |  |  |  |
| *E. pteroneura* | 0.3913043 | 0.4166667 | 0.4074074 | 0.2727273 | 0.3333333 | 0.3636364 | 0.0000000 |  |  |  |
| *E. royleana* | 0.3043478 | 0.3333333 | 0.2592593 | 0.2727273 | 0.3333333 | 0.4545455 | 0.3846154 | 0.0000000 |  |  |
| *E. smithii* | 0.3636364 | 0.3913043 | 0.3846154 | 0.3333333 | 0.3076923 | 0.4285714 | 0.3600000 | 0.3600000 | 0.0000000 |  |
| *E. tirucalli* | 0.3333333 | 0.3600000 | 0.2857143 | 0.3043478 | 0.2857143 | 0.3913043 | 0.3333333 | 0.2592593 | 0.2307692 | 0.0000000 |
| *Legend: 1=E. resinifera, 2=E. ampliphylla, 3=E. drupifera, 4=E. lathyris, 5=E. neoborascescens, 6=E. prostrata, 7=E. pteroneura, 8=E. royleana, 9=E. smithii, 10=E. Tirucalli*  **Supplementary Table 2**. Distribution of variable genes across chloroplast genome regions in 22 *Euphorbia* species   \| Category (Pi range) \| LSC \| SSC \| IR \| Total \| % of Total \| \| --- \| --- \| --- \| --- \| --- \| --- \| \| Highly variable (0.038 - 0.081) \| 29 \| 0 \| 4 \| 33 \| 25.6% \| \| Variable (0.024 - 0.037) \| 30 \| 0 \| 2 \| 32 \| 24.8% \| \| Conserved (0.012 - 0.024) \| 16 \| 0 \| 16 \| 32 \| 24.8% \| \| Highly conserved (0.000 - 0.012) \| 6 \| 12 \| 14 \| 32 \| 24.8% \| \| Total \| 81 \| 12 \| 36 \| 129 \| 100% \| | | | | | | | | | | |

**Supplementary Table 3.** Complete list of 129 chloroplast genes of 22 *Euphorbia* species organized by nucleotide diversity (Pi) and genomic region. IR genes are present in both IRa and IRb.

| **Category (Pi range)** | **Genes by Region** |
| --- | --- |
| **Highly variable (Pi = 0.038-0.081) 33 genes total** | **LSC (29 genes):**  *trnH-GUG, atpH, rpl14, psbT, rpl22, trnM-CAU, rpl36, rpl16, atpF, trnfM-CAU, psbB, petA, matK, trnS-GGA, trnC-GCA, rps4, psbI, accD, psaB, psbH, rps3, trnG-GCC, trnK-UUU, petB, psbK, rps14, atpI, atpE, atpA*  ***SSC (0 genes):***  *None*  **IR (4 gene entries - 2 unique genes):**  *trnV-GAC, rpl2* (each in IRa and IRb) |
| **Variable (Pi = 0.024-0.037) 32 genes total** | **LSC (30 genes):**  *trnP-UGG, psbZ, trnW-CCA, trnG-UCC, rps12, petG, petN, psbF, psbD, rpl20, psbJ, psbC, rbcL, petD, psbA, psbE, ycf3, rpoC2, atpB, rpoC1, ycf4, rps18, rpoA, clpP, rpoB, trnS-GCU, trnV-UAC, trnQ-UUG, psaA, psaI*  **SSC (0 genes):**  *None*  **IR (2 gene entries - 1 unique gene):**  rpl23 (in IRa and IRb) |
| **Conserved (Pi = 0.012-0.024) 32 genes total** | **LSC (16 genes):**  psbL, trnT-GGU, trnR-UCU, cemA, rpl33, trnL-UAA, ndhJ, rps2, trnD-GUC, trnT-UGU, rps8, trnE-UUC, psbN, trnF-GAA, ndhC, rps11  **SSC (0 genes):**  *None*  **IR (16 gene entries - 8 unique genes):**  *ndhB, ycf2, rrn16, rps19, rrn23, trnA-UGC, rps7, trnI-GAU* (each in IRa and IRb) |
| **Highly conserved (Pi = 0.000-0.012) 32 genes total** | **LSC (6 genes):**  *trnY-GUA, petL, psaJ, psbM, ndhK, trnS-UGA*  **SSC (12 genes):**  *ccsA, ndhD, ndhF, ndhH, trnL-UAG, ndhA, psaC, ndhG, rps15, ycf1, ndhE, ndhI*  **IR (14 gene entries - 7 unique genes):**  *trnL-CAA, rrn4.5, rrn5, trnR-ACG, ycf15, trnI-CAU, trnN-GUU* (each in IRa and IRb) |
